# Effects of acupuncture on patients with fibromyalgia: study protocol of a multicentre randomized controlled trial

**DOI:** 10.1186/1745-6215-12-59

**Published:** 2011-02-28

**Authors:** Jorge Vas, Manuela Modesto, Inmaculada Aguilar, Koldo Santos-Rey, Nicolás Benítez-Parejo, Francisco Rivas-Ruiz

**Affiliations:** 1 Pain Treatment Unit, "Doña Mercedes" Primary Health Care Centre, Dos Hermanas, Spain; 2 Support Research Unit, Costa del Sol Hospital, Marbella, Spain; 3 CIBER de Epidemiología y Salud Pública (CIBERESP), Spain

## Abstract

**Background:**

Fibromyalgia is a multidimensional disorder for which treatment as yet remains unsatisfactory. Studies of an acupuncture-based approach, despite its broad acceptance among patients and healthcare staff, have not produced sufficient evidence of its effectiveness in treating this syndrome. The present study aims to evaluate the effectiveness of individualized acupuncture for patients with fibromyalgia, with respect to reducing their pain and level of incapacity, and improving their quality of life.

**Methods/design:**

Randomized controlled multicentre study, with 156 outpatients, aged over 17 years, diagnosed with fibromyalgia according to American College of Rheumatology criteria, either alone or associated with severe depression, according to the criteria of the Diagnostic and Statistical Manual for Mental Disorders. The participants will be randomly assigned to receive either "True acupuncture" or "Sham acupuncture". They will be evaluated using a specific measurement system, constituted of the Fibromyalgia Impact Questionnaire and the Hamilton rating scale for depression. Also taken into consideration will be the clinical and subjective pain intensity, the patient's family structure and relationships, psychological aspects, quality of life, the duration of previous temporary disability, the consumption of antidepressant, analgesic and anti-inflammatory medication, and the potential effect of factors considered to be predictors of a poor prognosis. All these aspects will be examined by questionnaires and other suitably-validated instruments. The results obtained will be analysed at 10 weeks, and 6 and 12 months from the start of treatment.

**Discussion:**

This trial will utilize high quality trial methodologies in accordance with CONSORT guidelines. It may provide evidence for the effectiveness of acupuncture as a treatment for fibromyalgia either alone or associated with severe depression.

**Trial registration:**

ISRCTN trial number ISRCTN60217348 (19 October 2010)

## Background

The etiopathogeny of fibromyalgia syndrome (FMS) remains unknown, although current hypotheses centre on anomalous peripheral nociception caused by wind-up, central sensitivization, high levels of substance P and neurotrophins, and alterations to the hypothalamus-hypophysis-adrenal axis [1,2].

It is a multidimensional disorder, with currently poor therapeutic results. Despite the considerable increase in the number of studies published from 2000 to 2010, current treatment protocols are still unable to resolve the persistent symptoms experienced or improve the functional limitations and quality of life of these patients [3].

The prevalence of FMS among the Spanish population has been estimated at 2.7%, but at 4.2% for women and 0.2% for men [4]. Factors that may raise the risk of FMS include middle age, early school leaving and low family income.

Levels of anxiety and depression among patients with musculoskeletal pain are known to be related to FMS [5]; thus, the prevalence of patients with FMS and severe depression varies from 20-80% [6].

Pharmacological treatment continues to be the chief treatment option; in this respect, an important role is played by tricyclic antidepressants, which have a direct effect on the reuptake of serotonin and norepinephrine, thus improving sleep patterns and alleviating depression, stress and anxiety, as well as inhibiting pain pathways and recognition [7], although they have only been proved to be moderately effective [8,9]. Studies with new dual serotonin-norepinephrine reuptake inhibitors have produced promising preliminary results [10-12]. In addition, pregabalin has produced improvements, in comparison to a placebo, with respect to the treatment of pain, asthenia and sleep disorders among patients with FMS [13], and the combination of paracetamol and tramadol has also been found to be beneficial [14,15]. These approaches, thus, are opening up new possibilities in the pharmacological treatment of FMS syndrome. New contributions to our understanding of the etiopathogenic mechanism of FMS are orienting treatment toward improving central sensitivization, for example via antagonists of N-Methyl-D-aspartate (NMDA) receptors [16].

Non-pharmacological options include aerobic exercise, and muscle toning and stretching [3,17], which activate anti-nociceptive mechanisms and achieve pain reduction. There is moderate evidence that aerobic exercise is more beneficial than flexibilization, but no evidence that any one type of exercise is superior to another [18]. Cognitive-behavioural therapy has proved to be effective for alleviating symptoms and pain-related behavioural disorders, by improving central sensitization and activating anti-nociceptive mechanisms [19,20].

Acupuncture has been used as a treatment option in China for over 2000 years [21] and is increasingly accepted in the West, where its use has become considerably more common in recent decades, especially for pathologies producing high levels of pain [22,23], and thus it has been suggested as a remedy for FMS [23,24].

According to traditional Chinese medicine, FMS results from an imbalance that blocks or exhausts a person's internal energy (*Qi*) and the flow of blood, giving rise to the appearance of the symptoms that are characteristic of this syndrome [25,26].

Despite the broad acceptance of acupuncture among patients and healthcare staff, the studies conducted to date have not produced sufficient evidence of its effectiveness in treating FMS [27], although the latest systematic reviews have shown these studies to be of low quality [17,28,29]. Since the last of these published reviews, in June 2004 [27], various other studies, of higher quality, have been conducted, but the results they report are uneven, and thus little light is shed upon the role of acupuncture in treating FMS. One well-designed study [30] randomised 100 FMS patients into among four groups (one given true acupuncture, and the other three, sham acupuncture), with two sessions per week being given for 12 weeks. No differences were found among any of the outcome measures, but this is not surprising, as the authors used a standard prescription of acupuncture points, which is not the correct procedure [31]. Similar results have been found in another study, also well designed, which concluded that the level of analgesia attained is independent of the location of the acupuncture needles [32]. On the contrary, another well-designed study [33] obtained positive results on comparing real acupuncture with a placebo in terms of relieving pain, asthenia and anxiety, with a reduction of 7 points on the scale of the Fibromyalgia Impact Questionnaire. Another study, carried out in 2008, also reported a reduction in pain intensity and an improvement in quality of life, three months after acupuncture treatment was applied to a group of FMS patients, in comparison with tricyclic antidepressant treatment and exercise [34]. Acupuncture appears to be both safe and effective in treating depression, and is comparable with antidepressant treatment [35].

In view of these data, we designed this randomized controlled multicentre study of FMS patients, with the aim of determining the effectiveness of traditional acupuncture, using a point-selection algorithm established on the basis of the particular characteristics of each patient (seeking to reproduce standard clinical practice), and evaluating the progression of the illness using a specific measurement system, following OMERACT recommendations [36], and evaluating levels of depression, clinical and subjective pain intensity, the family life cycle, psychological aspects, quality of life, the duration of short-term disability, the consumption of anti-depressant, analgesic and anti-inflammatory medication, and the potential effect of factors considered to be predictors of a poor prognosis [37].

## Methods

### Trial objectives

The primary objective of the trial is to evaluate the effectiveness, in terms of pain reduction (measured on a 0-100 mm visual analogue scale) achieved at 10 weeks after beginning treatment. The secondary objectives of the trial are 1) to evaluate the effectiveness, in terms of reducing levels of depression (measured on the Hamilton scale, HAMD) at 10 weeks and 6 months after beginning treatment; 2) to evaluate the effectiveness, in terms of improvement measured by the Fibromyalgia Impact Questionnaire (FIQ), at 10 weeks, and 6 and 12 months after beginning treatment, with respect to both the overall value and the subscales of physical function, tiredness, depression and anxiety; 3) to evaluate the effectiveness in terms of reduced pain intensity (measured on a 0-100 mm visual analogue scale) at 6 and 12 months after beginning treatment; 4) to evaluate the effectiveness in terms of improvement perceived by the FMS patient at the end of the treatment; 5) to evaluate the effects on duration of the incapacity for work; 6) to analyse the number and the threshold of tender points perceived by FMS patients, and the changes in this respect after treatment; 7) to evaluate the effectiveness in terms of reduced consumption of anti-depressant, analgesic and anti-inflammatory medication; 8) to describe the family structure, the relation among family members and the family life cycle of FMS patients (genogram); 9) to evaluate the effectiveness in terms of improved health-related quality of life (SF 12); 10) to evaluate the cost-benefit aspect of acupuncture treatment for FMS patients.

### Hypothesis

Acupuncture is capable of reducing the pain felt by patients with fibromyalgia (FMS), whether in simple form or associated with severe depression, to a greater degree than is sham acupuncture. Furthermore, the application of this technique raises the patient's sense of wellbeing, reduces levels of depression, alleviates dysfunction, enhances health-related quality of life and moderates the consumption of drugs used in conventional treatment, thus reducing the negative effects produced by treatment without itself producing any clinically important iatrogeny.

### Design

**Figure 1:**
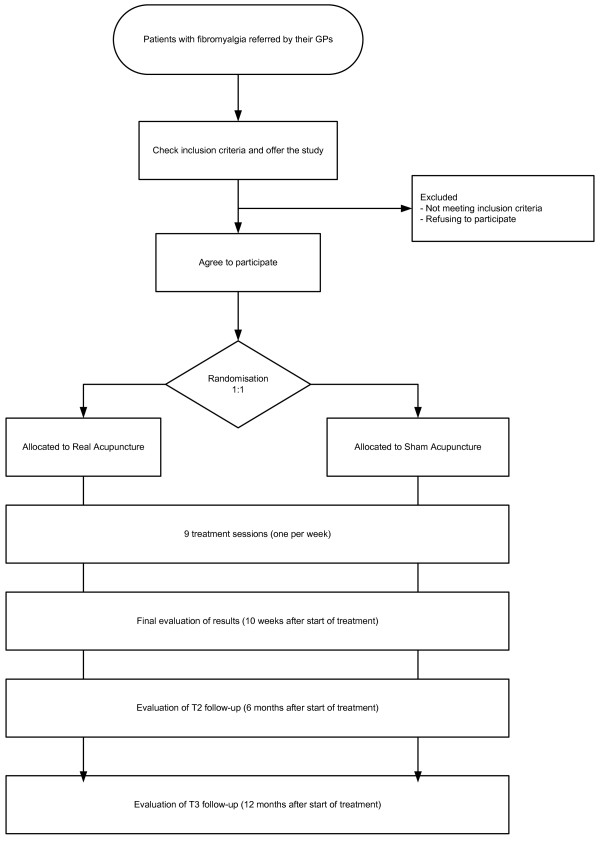
**Flow diagram for the study**. Work scheme with description of assessment visits and times.

Controlled multicentre prospective study, with random assignation to receive individualized acupuncture (according to traditional practice and individual diagnosis) or sham acupuncture (control group) with a 1:1 allocation ratio (Figure 1). Patients will be stratified according to level of depression and by treatment centre, and will be blinded to both types of treatment. The evaluation of patients and the analysis of results will be performed by professionals blinded to the assignation of treatment options.

### Study period

October 2010 - December 2013. At present, no patients have been recruited.

### Setting and participants

Outpatients, aged over 17 years, referred by their GPs to one of three primary healthcare centres participating in the study, and belonging to the Andalusian Public Health System. These will be patients diagnosed with FMS according to the criteria of the American College of Rheumatology (ACR) [38], and who have not previously received acupuncture treatment. The Hamilton scale (HAMD) will be used to stratify the patients into two sub-groups (cut-off point: 21), with or without severe depression.

The patients will be informed as follows: "This study will compare two types of acupuncture. One of them is similar to traditional Chinese acupuncture. The other does not follow these principles, but both types have been associated with positive results in different clinical studies". The patients will also be informed of the possible risks associated with the different types of acupuncture (infection, fainting or bruising) and that they may conclude their participation in the study at any time, without suffering any penalty or loss of benefits to which they would otherwise be entitled.

Any patient suffering pain for any reason other than FMS, or using anticoagulants or opiates, or who is pregnant or a nursing mother, or who is involved in occupational litigation for reasons involving FMS, will be excluded from this study.

### Sample size

The sample size is set at n = 56 patients in each group, for a significance level of 5%, a power of 90% and a difference in the mean pain intensity - according to 0-100 mm VAS - between the real and the sham acupuncture groups of 20.2 points, assuming equal variances (standard deviation = 32.4 points), taking into account the results of an earlier pilot experiment, carried out from April to December 2008. Assuming a dropout rate of 25%, the sample size will thus be n = 75 for each group (n = 78 to adjust by strata). These calculations were performed using the SamplePower 2 program [39].

### Recruitment of patients

The patients are referred by their GPs, in the areas where the study is performed. We have designed a data-compilation form containing the variables of interest, and this will be completed by the corresponding researcher at each centre. At each centre, the information obtained will be recorded on an electronic database, for subsequent statistical analysis.

### Randomisation

The randomisation of the two branches of this study will be performed in a centralised way, allocating the participants with stratification by centre and depression diagnosis and blocking following a 1:1 allocation ratio. The healthcare professionals participating in the study will not take part in the randomisation process. Those patients who meet the criteria for inclusion and who provide written informed consent will be included in the study. Following their inclusion, the researcher will contact the randomisation centre where the patient is registered, and the physician will be informed, by telephone and by fax, of the patient's assignation to one of the two study branches. This procedure ensures that the randomisation is not influenced by the researchers taking part in this study.

### Treatment

The patients taking part in the study will each receive nine acupuncture sessions (one per week), either real or sham, as follows:

a) **Real acupuncture**: Individualised acupuncture, using single-use sterile needles of diverse calibres and lengths. Guidelines have been agreed regarding the selection of points (Table 1). The physician will perform a prior diagnosis, in accordance with the principles of Traditional Chinese Medicine, before selecting the points for the treatment session, taking note of the diagnosis, the acupuncture points selected and the techniques employed.

**Table 1:** Procedure agreed upon for the selection of acupuncture points*

Basic syndrome	Supine	Prone	
Liver-Spleen disharmony	LI4 ↓	BL18 ↓	
	PC6 ↓	BL20 ↑	
	SP6 ↑	SP6 ↑	
	LR3 ↓	GB34 →	

**Associated syndrome**			**Left/Right**

Yin Deficiency and Empty Heat	Add KI6	Add BL23	

Spleen and Kidney Yang Deficiency	Add ST36	Add BL23	

Kidney Yin and Yang Deficiency	Add CV6	Add 52V	

Rising Liver Fire	Replace LR3 with LR2	Replace BL18 with LR2	

Phlegm	Add ST40 CV12	Add BL21	

Wet Heat	Add SP9	Add LI11	

Stomach and intestines	Add ST25	Add BL25 or BL21	

Bladder	Add CV3	Add BL28	

Genitals	Add CV2	Add BL31	

Associated pruritis	Add LR5		

Blood Stasis	Add SP10	Add BL17	

**Additional symptoms**			

Anxiety	Yintang		

Depression	GV20		

Severe asthenia	ST36 SP3		

Irritability	PC6		

Insomnia	HT7		

Chest tightness	CV17 PC6		

Inhibited sexual desire	GV4		

Nicturia	BL52		

Pollakisuria	CV3		

Mushy stool	SP9		

Night sweats	HT6		

Severe generalised pain	SP21		

Occipital headache	GB20		

Vertex headache	GV20		

Temporoparietal headache	Taiyang		

Neck pain	GB20 GB21		

Back pain	SI12		

Brachialgia	LI10		

Lumbalgia	BL23 BL25		

Pain in the hip/trochanter	GB30 GB29		

*: Standard acupuncture points for treatment of these symptoms [31]

The treatment will be performed after sterilizing the skin on the areas where the needles will be inserted, and with the patient lying face up or face down. A vertical puncture will be made, unless otherwise indicated, to the depth predetermined for each point (normally between 8-30 mm, depending on the location of the point). Following insertion, stimulation of the acupuncture point will be performed using of bidirectional rotation of the needle sleeve, to achieve the sensation known as *Deqi*, which is commonly described as a 'glowing' feeling. The needle will be maintained in place for 20 minutes, with bidirectional rotation of the needle sleeve (with amplitude and rotation speed as stipulated previously) for one minute, every five minutes (a total of four such rotations per session). Following the treatment session, the needles will be withdrawn. Before each session, the physician will re-evaluate the patient to determine whether his/her clinical situation has changed; if so, the selection of acupuncture points will be reconsidered.

B) **Sham acupuncture**: With the patient lying face down, the insertion of needles into the back and lower back will be simulated. This is a technique, validated previously [40], in which, after sterilization of the surrounding skin, a momentary pressure is exerted using a plastic guide tube through the centre of which a blunt steel rod is inserted, producing the sensation that a puncture has been made, at each of the following points. This plastic guide tube will be presented in containers identical to those used for the real acupuncture group. The patient should remain face down for the 20 minutes of the session, so that the placebo technique remains concealed. Every five minutes, the physician applying the treatment will repeat the action at the corresponding eight points (which are not acupuncture points [31]):

1. Located bilaterally at 1 cm from the spinal apophysis of T3

2. Located bilaterally at 1 cm from the spinal apophysis of T5

3. Located bilaterally at 1 cm from the spinal apophysis of L2

4. Located bilaterally at 1 cm from the spinal apophysis of L5

The same time will be dedicated to the patients in each of the treatment groups; similarly, the time employed for the pre and post-session evaluations will be identical in every case. The healthcare personnel applying the different acupuncture treatments have received at least 300 hours training in the field and have over three years practical experience. All adverse reactions or side effects that may occur will be recorded in the Data Record Book, with a detailed account of the circumstances and the date of occurrence. No relative or other person accompanying the patients in this study will be allowed to enter the treatment room.

### Data collection

The data required for evaluating the effectiveness of the treatment will be collected at baseline and 10 weeks, 6 and 12 months after the intervention has begun, for both groups. Data will be obtained via interviews, self-applied questionnaires, and physical measurements. Data collection instruments and the study timeline are summarised in Table 2.

**Table 2:** Data collection instruments at different assessment points

Variable	T0	2s	3s	4s	5s	6s	7s	8s	9s	T1	T2	T3
*HAMD*	X									X	X	

*FIQ*	X									X	X	X

*0-100 mm VAS*	X									X	X	X

*SF12*	X									X	X	X

*Genogram*	X											

*Tender points count*	X									X	X	X

*Pain threshold*	X									X	X	X

*Improvement perceived by patient*										X		

*Expectations and credibility*			X							X		

*Side effects*										X		

*Diagnosis by traditional Chinese medicine*	X									X		

*Medication consumed*	X									X		

*Occupational data*	X									X	X	X

*Sociodemographic data*	X											

VAS: Pain intensity measured on a visual analogue scale; HAMD: Hamilton scale; FIQ: Fibromyalgia Impact Questionnaire; T0: Baseline value; T1: Final value (10 weeks after start of treatment); T2: Follow-up evaluation 1 (6 months after start of treatment); T3: Follow-up evaluation 2 (12 months after start of treatment); #s: Session number.

A data collection form has been designed, to include the variables of interest, which will be completed by the corresponding researcher at each centre. At each centre, the information obtained will be recorded on an electronic database, for subsequent statistical analysis.

The baseline values (T0), those for the results after 10 weeks (T1) and the follow-up results at 6 and 12 months (T2 and T3) will be determined by evaluators who are blinded to the treatment assignation groups. These same evaluators will provide any necessary assistance regarding the self-administered questionnaires. The Hamilton scale will be applied by specialists in the field, who will also be blinded to the treatment assignation groups.

### Outcomes

#### Primary outcome

Changes in pain intensity, measured on a visual analogue scale, at the end of treatment. The construction validity and reliability of this scale have been proven in previous studies [41-43]. The scale measures a continuous quantitative variable varying from 0 (absence of pain) to 100 (the worst pain imaginable).

#### Secondary outcomes

- Changes in levels of depression, measured on the Hamilton hetero-evaluation scale, at the end of treatment and after 6 months. This scale is of proven discriminant validity, reliability, and sensitivity to change; moreover, it has been validated for use in Spain [44]. It consists of 17 items. Each question has from three to five possible responses, with scores varying from 0-2 to 0-4, respectively. The total score varies from 0-52. To evaluate the response to treatment, a full response is defined as a reduction of 50% or more in the initial score obtained on the scale; a partial response is taken as a reduction of 25-49%, and absence of response is defined as a reduction of less than 25%. Remission is considered to have been achieved with a score of 7 or less, although according to some studies this cut-off point should be lower [45].

- Changes in the overall indicator value and in the different subscales of the Spanish version of the Fibromyalgia Impact Questionnaire [46], at the end of treatment (10 weeks after the start), and at 6 and 12 months from the start of treatment. This is a self-administered questionnaire that measures the aspects of health most affected by FMS during the previous week [47]. It is comprised of ten items, with three questions valued on a Likert scale, and a further seven questions valued on a visual analogue scale, varying from 0 to 10, with the higher scores reflecting a greater negative impact or more severe symptoms.

- Changes in pain intensity on a visual analogue scale, measured at the end of treatment, and at 6 and 12 months after beginning treatment.

- Changes in the pain threshold and number of tender points detected by experienced evaluators, assigned one per centre and non-interchangeable, using a digital pressure algometer with a contact point of 1 cm^2 ^(NIDEC SHIMPO - FGE-100 X - Digital Force Gauge). The pressure applied is 1 kg/s at each of the 18 tender points specified [48]. The patients will be asked to tell the researcher when the sensation of pressure changes to one of pain. From all of these pain points, a mean threshold will be calculated. Normal subjects begin to perceive pain at a pressure of 4 kg [38]. The baseline measurements will be compared with those obtained at the end of treatment, and after 6 and 12 months.

- Improvement perceived by the patient [49], measured on a seven-point Likert scale. "How satisfied are you with your recent treatment for fibromyalgia?" 1 = Extremely satisfied; 2 = Very satisfied; 3 = Moderately satisfied; 4 = Indifferent (an approximately equal degree of satisfaction and dissatisfaction); 5 = Moderately dissatisfied; 6 = Very dissatisfied; 7 = Extremely dissatisfied.

- Changes in patient's health-related quality of life, according to the SF-12 Version 2 Questionnaire. This is a generic questionnaire, derived from SF-36, and validated for Spain [50]. Version 2 enables the researcher to calculate the patient's quality of life in eight dimensions (physical function, physical role, pain, general health, vitality, social function, emotional role and mental health) and two summary components (physical and mental), scale 0-100; lower scores indicate poorer quality of life.

- Consumption of medication. Anti-depressant, analgesic and NSAIDs medication consumed (whether or not prescribed by the patient's doctor) at the moment of randomisation and at follow up (at each treatment session, at the end of treatment and at 6 and 12 months), measured on a four-point Likert scale: 0 = no medication; 1 = less than the usual dose; 2 = daily, at the usual dose; 3 = greater than the usual dose. A record will also be made, by the evaluator, of the names of the pharmaceutical preparations taken by the patient, and the daily dose.

- Expectations and credibility of the treatment [51]. These will be measured using an original scale devised by Borkovec and Nau [52], with four items measuring the following on a continuous visual analogue scale from 0 to 10 (0 = totally disagree, to 10 = totally agree): (1) Do you believe this treatment will alleviate the pain you feel?, (2)Do you believe this treatment to be a logical one?, (3) Would you recommend this treatment to a friend or relative suffering from the same problem?, (4) Do you believe this treatment would be a useful option for dealing with other problems? Items 1 and 2 will be evaluated after the second treatment session, and items 3 and 4 after the eighth session.

- Side effects and adverse reactions. A record will be made of the side effects and possible adverse reactions arising from the treatment.

### Covariables

- Family structure and relations, determined by the genogram technique [53].

- Perceived pain threshold and tolerance (count of positive tender points and determination of the pain threshold and tolerance using a pain meter).

- Sociodemographic variables: age, sex, race, education level, profession, income level, weight (kg), and height (cm).

- Dependence on tobacco, alcohol or other pyschoactive substances.

- Any present comorbidity.

- Diagnostic characteristic variables, according to traditional Chinese medicine.

### Data storage and confidentiality

All questionnaires are stored in a locked cabinet in a locked room at each participating centre, and have a unique identification number. Consent forms are stored separately from study questionnaires in a locked cabinet. Only anonymised data are entered into the computerised study database, and access to the database is restricted to the study team.

### Statistics

The statistical analysis will be carried out for two types of groups: (1) per intention to treat, with all patients randomised; (2) per protocol, including only patients presenting no more than minor deviations from the protocol.

We will compare the baseline variables for the different groups in terms of difference of the means and of proportions. The magnitude of the difference in the possible imbalance produced by the random assignation to the groups will be evaluated using ratios of the means and of proportions (using that of the control group (sham acupuncture) as a reference level, and the final adjustment will be made using secondary analyses with multiple linear regression models, as described below. In the unadjusted analysis, significance tests will be used to compare the sample (parametric or otherwise, depending on the symmetric or asymmetric distribution of the result variables and on the homogeneity of their variances), taking the control group as a reference, using comparison tests for differences of the mean in the main results variable, both as regards inter-group comparisons (for independent samples) and for comparisons between the baseline and final levels for each group (in this case, using tests for non-independent or paired samples).

For the main result variable, pain intensity, we will construct linear regression models, adjusted for baseline level, treatment centre and depression, and using intention to treat analysis. The group variables will be included, taking the control group as a reference, together with the sociodemographic variables (age and sex), together with the baseline variables for severity of the process (pain intensity, FIQ and HAMD) and pain threshold and tolerance. Adjustments will be made for possible confounders, using criteria of statistical significance and confounding. The detection of possible interactions with the treatment group variable will be evaluated using statistical significant criteria for the corresponding interaction terms. P values < 0.05 will be declared to be statistically significant.

For the cost effectiveness analysis, a non-parametric bootstrapping approach will be used with the incremental cost effectiveness preference map to illustrate the willingness to accept: willingness to pay ratio, and a 95% confidence interval will be computed for the different measures of effectiveness [54]. For cost imputation, drug consumption will be taken into account, and different thresholds established for acceptance of the experimental treatment, to evaluate diverse scenarios. For the cost associated with the treatment, the all patients Diagnosis Group Relationship measure will be used, in association with the main diagnosis, together with the average professional salary/hour, and the indirect costs associated with acupuncture sessions.

### Ethical considerations

The ethical validity of this study has been corroborated by the Andalusian Committee for Clinical Trials, following approval by the Research Committee at the Valme Hospital (Sevilla, Spain). It will be carried out in accordance with the Helsinki Declaration and its subsequent amendments, up to and including the 2008 review [55], taking into account the principles set out in the Convention of the Council of Europe on Human Rights and Biomedicine [56], as well as the requirements imposed by Spanish legislation with respect to biomedical research, personal data protection and bioethics [57]. All the patients taking part must give their written informed consent to the clinical research procedures proposed. During this study, audits will be performed as considered necessary by the corresponding research ethics commission, as well as the Hospital's own Quality Commission, independently of any external audits (research funders) that may be called for. The statistical analysis will be carried out by third parties who will be unaware of the origin of the data (blind analysis).

## Discussion

When acupuncture is practised in accordance with the principles of Traditional Chinese Medicine (TCM), it is an individualised therapy. In this study, we have designed a treatment protocol resembling standard clinical practice, one that is sufficiently flexible as to address individual variability in a reproducible form. Nevertheless, the study is an experimental one in which the acupuncture treatment cannot be implemented to its full extent. Unlike standard clinical practice, a semi-standardised protocol must be followed, so that the study may be replicated, and the number of points where acupuncture is applied must be limited. This may mean that the effect obtained is inferior to that expected in standard practice, although we have designed a point selection consensus algorithm to cover the clinical presentations most commonly adopted. This limitation does produce an effect contrary to the study hypothesis, although the design of a consensus algorithm will tend to reduce its impact. On the other hand, the control branch of the study, due to the attention provided and the effects of peripheral sensory stimulation, however minimal they may be, could have some positive results. This fact, too, is contrary to the study hypothesis, but to an extent it reflects the non-specific effect of the intervention.

It is not possible to perform a double-blind study, because the acupuncture practitioner must know what treatment is being applied. We attempt to overcome this problem by preventing the practitioner from performing the evaluation of outcome measures, as well as ensuring that the blinding is maintained of both the evaluators and the patients.

One important limitation that may be present in this study is the possible non-adherence of patients to the treatment prescribed, as for various reasons they may fail to attend any given treatment session. The main study analysis was per intention-to-treat, but a per protocol analysis was also performed. The study size was calculated on the assumption of a 25% loss rate, but it will be necessary to ascertain that no differential losses between the two treatment branches take place.

This trial will utilize high quality trial methodologies in accordance with CONSORT guidelines [58]. It may provide evidence for the effectiveness of acupuncture as a treatment for fibromyalgia either alone or associated with severe depression.

## Competing interests

The authors declare that they have no competing interests.

## Authors' contributions

JV conceived the study, designed the study protocol, sought funding and ethical approval and wrote the manuscript. MM, IA and KS made a substantial contribution to designing the individualised acupuncture treatment protocol. NB and FR are responsible for the statistical analyses. All authors have critically reviewed and approved the final version of the manuscript. The corresponding author had final responsibility for the decision to submit for publication.
